# Gastrointestinal Stromal Tumor (GIST) Masquerading as a Pancreatic Pseudocyst: A Rare Case Report

**DOI:** 10.7759/cureus.66491

**Published:** 2024-08-09

**Authors:** Sarthak Sharma, Varun Shetty, Iqbal M Ali

**Affiliations:** 1 General Surgery, Dr. D.Y. Patil Medical College Hospital and Research Centre, Dr. D. Y. Patil Vidyapeeth, Pimpri, IND

**Keywords:** gastrointestinal stromal tumour(gist), rare, mesenchymal tumors, solid tumors, epithelioid

## Abstract

Despite their relative rarity, gastrointestinal stromal tumors (GIST) are the most common type of mesenchymal tumor in the gastrointestinal (GI) tract. Here, we describe a rare case of a 62-year-old hypertensive female presenting with abdominal pain and a palpable mass, initially suspected to be a pancreatic pseudocyst based on radiological findings. Subsequent histopathological (HPE) examination following surgical resection revealed a large cystic lesion originating from the stomach, characterized as a malignant epithelioid GIST. Based on these findings and taking into consideration the symptomatology of the patient, the decision was made to post the patient for an upfront, open surgical exploration without pre-operative biopsy studies. Frozen section facilities were kept on standby considering the differential diagnosis. Since the frozen section revealed a gastric GIST, a decision was made to perform subtotal gastrectomy, followed by gastrojejunostomy (GJ) and jejunojejunostomy (JJ). In addition, the part of the cyst adherent to the left lobe of the liver was dealt with with a non-anatomical wedge resection. Immunohistochemical (IHC) analysis showed positivity for Cluster of Differentiation 117 (CD117) with negativity for Cluster of Differentiation 34 (CD34), Desmin, and Discovered On Gastrointestinal Stromal Tumors 1 (DOG-1). The tumor exhibited aggressive features, including high mitotic activity, i.e., >5/10 high power field (hpf), hemorrhagic areas, and infiltration into the liver parenchyma. The patient then received adjuvant imatinib-based chemotherapy and was maintained on strict follow-up.

## Introduction

Gastrointestinal stromal tumors (GIST) are relatively rare but represent the most common type of mesenchymal tumor found in the gastrointestinal (GI) tract [1]. These tumors primarily affect the stomach, accounting for approximately 60% of cases, and the small bowel, making up about 30% [2]. GIST typically occurs in individuals between the ages of 50 and 70. Symptoms can vary widely, depending on the site of origin. The common clinical presentation often includes a lump in the abdomen associated with vague abdominal pain. Occasionally, gastric GIST can present as gastric ulcers, gastro-duodenal bleeding, and melena [3]. In some cases, GIST is discovered incidentally during radiological studies performed for other reasons [3,4].

Histologically, GISTs are characterized by their solid tumor presentation and can exhibit diverse growth patterns. Histologically, about 70% of these tumors have fusiform-shaped cells, 20% present with an epithelioid cell pattern, and 10% show a mixed cellular pattern [5]. The solid nature of GIST is a defining feature, and they rarely exhibit cystic changes, especially if the tumor is large. These histological characteristics are crucial for the diagnosis and differentiation of GIST from other types of tumors that may occur in the gastrointestinal tract [6].

The clinical presentation and imaging features of GIST can sometimes make diagnosis challenging. While solid tumors are typical, the presence of cystic changes, although rare, can complicate the diagnostic process. A comprehensive radiological and endoscopic evaluation, accompanied by a biopsy and histological examination, is essential for an accurate diagnosis. However, despite extensive pre-operative evaluation, some cases of GIST can have an abstract presentation, as in our case. Recognizing the varied histological patterns and understanding the common and uncommon presentations of GIST can aid clinicians in timely and accurate diagnosis, ensuring appropriate management and treatment for affected patients.

## Case presentation

A 62-year-old hypertensive female presented with a two-month history of abdominal pain and malaise, which had worsened over the past 2-3 days. The pain was mainly limited to the epigastric region and was dull and aching in nature, aggravated post-meals. It was associated with occasional vomiting containing food particles. She gave a history of similar episodes of pain in the abdomen, on and off, over the previous year. She reported noticing a mass in her abdomen for 1.5 months, accompanied by a burning sensation in the epigastric region and a reduced appetite. The patient had no prior history of abdominal surgery, hematemesis, melena, altered bowel habits, fever, jaundice, trauma, or weight loss.

The general examination findings were within normal limits. Upon physical examination, a tender lump approximately 10 by 12 centimeters was palpable, involving the epigastric and left hypochondrium regions. The lump had diffuse margins and was firm on palpation. On percussion, however, the lump had a resonant note. Succussion splash was absent. The rest of the abdominal examination was unremarkable, with no organomegaly or free fluid in the abdomen. All vital signs were stable. The other systemic examinations were also unremarkable. Key laboratory investigations, including renal function tests (RFT), liver function tests (LFT), cancer antigen 19-9 (CA-19.9), carcino-embryonic antigen (CEA), and serum amylase, were within normal limits, but serum lipase was elevated at 950 U/L (Table 1).

**Table 1:** Laboratory parameters were observed preoperatively

INVESTIGATION	OBSERVED PARAMETERS	REFERENCE INTERVAL (AS PER OUR INSTITUTIONAL LABORATORY)
Total Bilirubin	1.1 mg/dl	0.22-1.20 mg/dl
Serum Glutamic-Oxaloacetic Transaminase (SGOT)	42 U/L	8-48 U/L
Serum Glutamate Pyruvate Transaminase (SGPT)	40 U/L	7-55 U/L
Alkaline Phosphatase	121 U/L	40-129 U/L
C-Reactive Protein (CRP)	3.8 mg/L	Up to 5.0 mg/L
Serum urea	40 mg/dl	17-49 mg/dl
Serum Creatinine	1.1 mg/dl	0.6-1.3 mg/dl
Hemoglobin (Hb)	13.2 g/dl	13.2-16.6mg/dl
Total Leukocyte Count (TLC)	6800/ uL	4000-10000/uL
Cancer antigen (CA 19.9)	4 U/ml	Less than 37 U/ml
CEA	1.1 ng/ml	0-3 ng/ml
Serum Amylase	42 U/L	40-140 U/L
Serum Lipase	950 U/L	50-120 U/L

**Figure 1:**
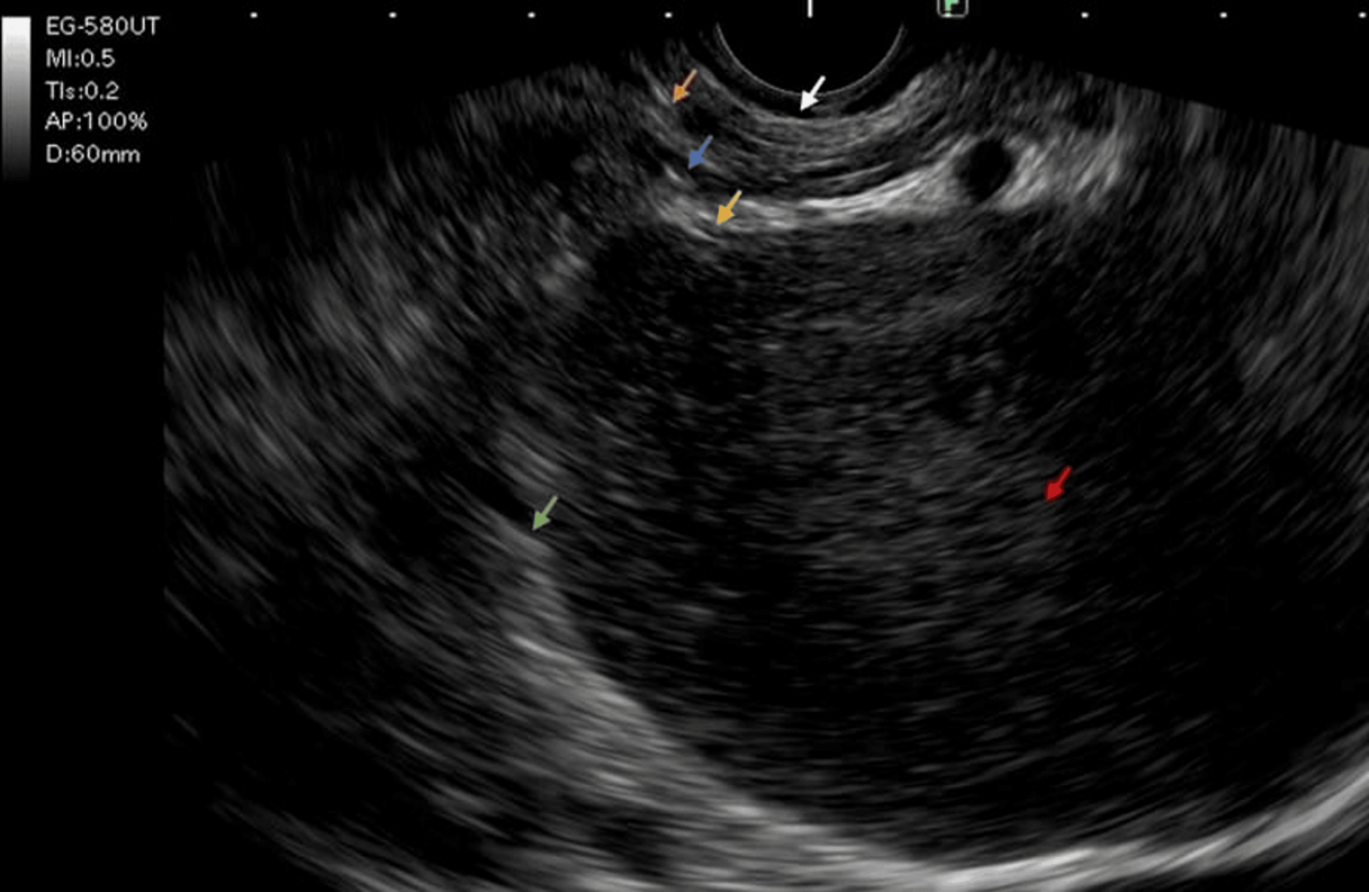
Endoscopic ultrasound (EUS) image showing the cystic lesion in relation to the stomach (pseudocyst of the pancreas/gastric GIST) White arrow: Superficial mucosal layer of the stomach, Orange arrow: Deep mucosa of the stomach, Blue arrow: Submucosal layer of the stomach, Yellow arrow: Muscularis propria of the stomach, Green arrow: Serosa of the stomach, Red arrow: a cystic lesion of size 10*12*10 cm with a wall thickness of 7 mm without any septations. The lesion appears to be related only to the serosal layer of the stomach.

Esophagogastroduodenoscopy (OGD) indicated an extrinsic impression at the body antrum, suggestive of a pancreatic pseudocyst with compromised gastric lumen. However, the scope could be negotiated into the duodenum. Endoscopic ultrasonography (EUS) revealed a cystic lesion that appeared to be arising from the pancreatic body and was seen abutting the wall of the stomach, suggestive of a cystic neoplasm, pancreatic pseudocyst, or hydatid cyst. Although the findings of EUS were not conclusive in terms of the site of origin, a provisional diagnosis of a pancreatic pseudocyst was considered (Figure 1). The prior history of similar complaints, suggestive of pancreatitis, also played a role in the decision-making process. 

**Figure 2:**
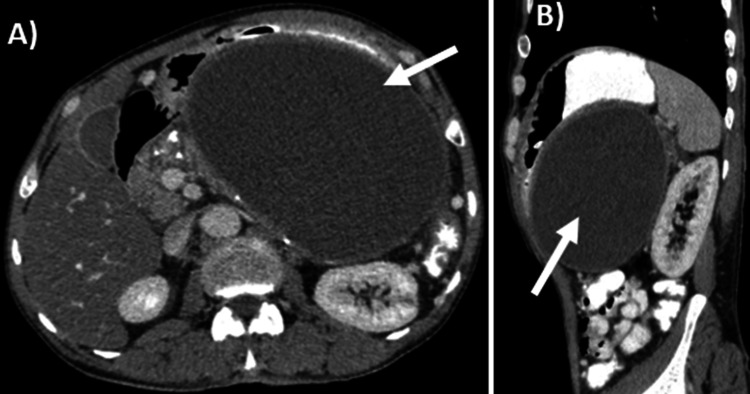
CECT: abdomen and pelvis (axial and sagittal cuts) demonstrating a predominantly cystic lesion compressing the stomach and left lobe of the liver A) Axial cut: CECT (Abdomen): Portal venous phase A large, well-defined, hypodense, uniformly cystic lesion of size 136*117*122 mm (white arrow) is seen arising from the body of the pancreas, causing severe compression of the gastric antrum. It is seen extending in the gastrohepatic ligament between the stomach and left hepatic lobe, showing a peripheral solid component. The pancreas appeared bulky, with no per-pancreatic fat stranding. No obvious communication was noted with the main pancreatic duct (MPD). B) Sagittal cut: CECT (Abdomen): Portal venous phase A fairly large cystic, hypodense lesion occupying the lesser sac was seen abutting and causing compression of the gastric antrum. It showed peripheral enhancement in relation to the left lobe of the liver, indicating a solid component; however, with preserved fat planes. There is no associated lymphadenopathy. Findings were suggestive of a pseudocyst of the pancreas or gastric GIST. CECT: Contrast-enhanced computed tomography

Hydatid serology was negative for *Echinococcus* (hydatid cyst) and immunoglobulin G (IgG). Contrast-enhanced computed tomography (CECT) of the abdomen and pelvis showed a large, well-defined, uniformly hypodense cystic lesion measuring 136x117x122 mm in the epigastric region, causing severe compression of the gastric antrum and extending into the gastrohepatic ligament between the stomach and the left hepatic lobe. This lesion had a peripheral solid component, most likely indicating a pancreatic pseudocyst. The cyst wall had variable thickness, with a maximum thickness of 8 mm at the gastric antrum (Figure 2). 

Based on the radiological and endoscopic findings, a provisional diagnosis of pseudocyst of the pancreas or gastric GIST was made. However, we were biased towards a pancreatic pseudocyst based on the history and findings of the endoscopy. Based on these findings and taking into consideration the symptomatology of the patient, the decision was made to post the patient for an upfront open surgical exploration without pre-operative biopsy studies. Frozen section facilities were kept on standby considering the differential diagnosis.

On exploration through a midline laparotomy incision, a large cystic lesion was identified, almost obliterating the lesser sac. It was seen extending superiorly to the left lobe of the liver, where it was adherent. Laterally, it was seen extending up to the splenic hilum while the antrum of the stomach was compressed by the lesion. However, the lesion was seen to be arising from the posterior wall of the gastric antrum instead of the pancreatic body (as suggested by the radiology scans). No obvious macroscopic evidence of solid organ metastasis was noted on exploration. The cyst was decompressed, and an intraluminal examination was carried out, which showed a solid component involving the posterior wall of the gastric antrum. The fluid aspirated from the cyst was hemorrhagic without any necrotic material, which further raised the suspicion of a malignant lesion rather than a pseudocyst.

With a high level of suspicion for gastric GIST (as preoperatively contemplated), a biopsy from the suspicious gastric wall was sent for frozen section analysis. The preliminary evaluation revealed a malignant GIST arising from the gastric wall with a high nucleo-cytoplasmic (N/C) ratio and prominent nucleoli. Taking into account the frozen section findings as well as the local extension of the illness, a decision was made to perform subtotal gastrectomy, followed by gastrojejunostomy (GJ) and jejunojejunostomy (JJ). In addition, the part of the cyst adherent to the left lobe of the liver was dealt with with a non-anatomical wedge resection. The aspirated fluid was sent for biochemical evaluation, which revealed an elevated CEA level (18 ng/ml) with normal fluid amylase levels (<3 times serum amylase level).

**Figure 3:**
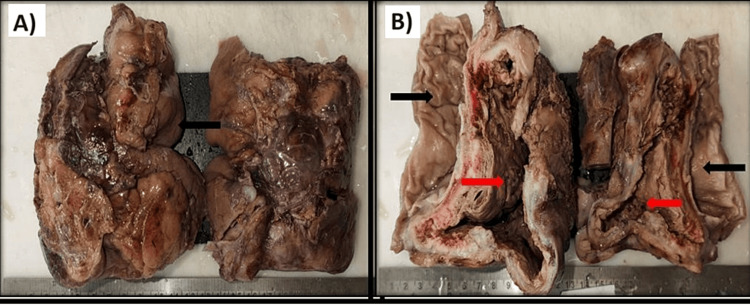
Gross pathology images 3A) Gross specimen showing the external surface of the cyst wall, stomach, and a part of the liver attached (black arrow). 3B) Section of a large, thick-walled cyst with irregular cyst wall thickness (red arrow) and the part of the unremarkable gastric mucosa (black arrow).

The excised specimen included a large cystic mass measuring 14x10x10 cm, attached to the stomach wall and filled with hemorrhagic fluid, along with a wedge from the left lobe of the liver (Figures 3A, 3B). The cyst wall was solid, brownish, necrotic, and irregular. The gastric mucosal surface was unremarkable, and the cyst wall was seen adhering to a resected wedge off the left lobe of the liver. Microscopy revealed a malignant tumor consisting of clusters of pleomorphic epithelioid cells with vesicular nuclei, prominent nucleoli, and increased mitosis, i.e., greater than 5/10 high power field (hpf). The tumor showed areas of hemorrhage and necrosis and was found to arise from the smooth muscle coat of the stomach without involving the gastric mucosa, infiltrating the liver parenchyma. The proximal and distal resection margins of the stomach were tumor-free, the resected liver margin was tumor-free, and two small lymph nodes were identified as tumor-free. 

**Figure 4:**
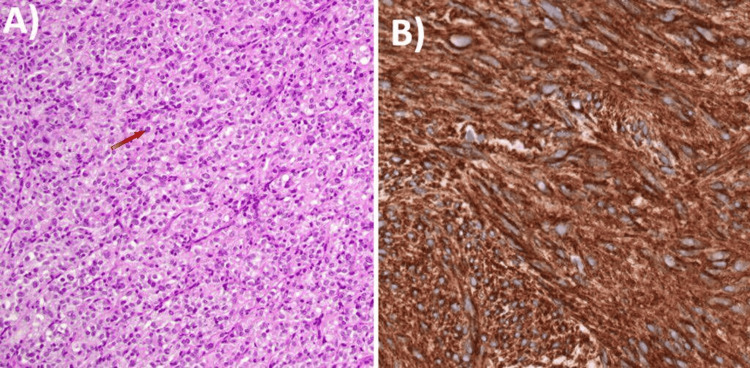
Histopathological (HPE) and immunohistochemical (IHC) staining images A) Malignant tumor comprising pleomorphic epithelioid cells (red arow) having vesicular nuclei, prominent nucleoli at places, and increased mitosis with areas of necrosis. B) CD117 immunoreactivity in GISTs that is strongly and uniformly positive in a cytoplasmic pattern

Immuno-histochemical (IHC) staining showed positivity for Cluster of Differentiation (CD117), with negativity for CD34, Vimentin, Beta-Human Chorionic Gonadotrophin (beta-HCG), Discovered on Gastrointestinal Stromal Tumor-1 (DOG-1), and Desmin markers. The marker of proliferation, Kiel 67 (Ki-67), indicated more than 20% proliferative activity (Figures 4A, 4B). 

Based on the intraoperative as well as postoperative findings and utilizing the modified NIH-Fletcher classification system for GIST, a final diagnosis was made. The main parameters that formed the basis of this classification included tumor size, mitotic index, site of origin, and tumor rupture (Table 2). This system categorizes tumor risk based on tumor size, mitotic index, and primary tumor site, with categories ranging from very low risk to high risk. 

**Table 2:** Modified NIH-Fletcher classification system for GIST NIH: National Institute of Health

Risk category	Tumor size (in cm)	Mitotic index (per 50 high power field)	Site of tumor origin
Very low risk	<2.0	Any	Any
Low risk	2.1-5.0	<5	Any
Intermediate risk	5.1-10.0	<5	Gastric
High risk	>10.0	Any	Tumor rupture
	Any	>10	Any
	>5.0	>5	Gastric
	<5.0	>5	Non-gastric
	>5.0	<5	Non-gastric

Accordingly, our case was staged as a malignant epithelioid variant of GIST with a high risk of recurrence (tumor size >10 cm with a high mitotic index of >10/50 hpf and gastric origin without rupture). Radiotherapy was advised to the patient in conjunction with imatinib chemotherapy based on the high risk of recurrence. However, the patient had refused to undergo radiotherapy due to financial reasons. The patient was then started on imatinib-based chemotherapy (400 mg orally for 12 months). The patient was advised to have regular, three-monthly follow-ups for the first year. On her first follow-up after three months post-surgery, the patient had no symptoms suggestive of complications or recurrence. Further follow-up is planned with a positron emission tomography (PET) scan after another three months. Informed consent was obtained from the patient for publication and research purposes.

## Discussion

GIST are the most commonly encountered mesenchymal neoplasms originating from the smooth muscles of the GI tract, typically positive for CD 117, and accounting for around 3% of gastric neoplasms [3,7]. They most often occur in individuals aged 50-70 years, presenting with symptoms such as GI bleeding, melena, gastric ulceration, abdominal pain, or as incidental findings on radiological imaging studies. GISTs are usually solid tumors, with cystic changes being a rare occurrence [8]. Only a few cases of such atypical presentations are reported in the literature [9-11]. In this case, the diagnosis of GIST was not clear preoperatively due to the lack of specific GI symptoms. Radiological investigations, including abdominal sonography, CECT, and gastric endoscopy/endoscopic ultrasound, suggested a pancreatic pseudocyst or gastric GIST but did not provide a definitive diagnosis.

A predominantly cystic transformation of GIST could be explained by the following scenarios, as explained by Bechtold et al.: (a) A primary GIST with a relatively high growth rate resulting in cystic transformation (b) distant metastasis from primary GIST presenting as cystic lesions; (c) primary predominantly solid GIST receiving adjuvant imatinib chemotherapy, resulting in cystic degeneration [12]. The infrequent occurrence of c-kit/PDGFRα mutations in these tumors may be attributed to a scarcity of neoplastic cells or could be a distinct inherent characteristic linked to their formation.

GISTs are primarily located in the stomach, followed by the small intestine, and the risk of metastasis increases with tumor size, particularly when larger than 10 cm [13]. Cystic changes are more common in malignant, high-grade GISTs, as seen in a case report described by Okano H. et al. [10]. The published case report demonstrated a high-grade GIST (size >5 cm with a mitotic index >5/50 hpf and gastric origin) with predominant cystic changes, similar to ours. Aggressive tumor growth and degenerative changes contribute to the formation of large cystic spaces [14]. In the Asian population, mixed spindle-epithelioid histology is commonly observed [15]. In the present case, the large tumor was located in the epigastric region, in relation to the lesser curvature of the stomach, extending into the gastro-hepatic ligament and infiltrating the liver parenchyma. The tumor was identified as an aggressive, malignant epithelioid cell type GIST with high mitotic activity (>5/10 hpf), which was in concurrence with the existing literature [13-15].

Tumors with spindle cell morphology and CD117 positivity are typically less aggressive [12]. Epithelioid patterns with platelet-derived growth factor receptor α (PDGFRA) mutations tend to have a favorable prognosis, whereas epithelioid or mixed patterns without PDGFRA mutations, particularly in stomach GIST, have an unfavorable course. These histological patterns (epithelioid and mixed) often express PDGFRA and sometimes CD34 [16,17]. On immunohistochemistry (IHC), tyrosine kinase receptor (KIT)-mutated tumors show CD117 positivity and respond to tyrosine kinase receptor (KIT) inhibitor therapy. Ki-67 correlates with the mitotic rate and prognosis. DOG1 is emerging as a promising biomarker for PDGFRA-mutated epithelioid GIST with weak or negative KIT expression [18]. Vimentin and Desmin are also positive in most cases; however, vimentin in our case was negative. In this case, the clinico-radiological picture suggested a pancreatic pseudocyst/gastric GIST, but intraoperative frozen section and final histopathology confirmed the diagnosis of a malignant epithelioid cystic GIST (high grade as per the modified NIH GIST grading system), with IHC markers showing positivity for CD117 while CD34, Desmin, and DOG-1 were negative, making the case atypical [18,19].

Pre-operatively, the following parameters could be utilized to distinguish between a pancreatic pseudocyst and a GIST (Table 3). These findings could help in better preoperative planning of the management and biopsy assessment of malignancy, thereby allowing for a more tailored and targeted therapy [19].

**Table 3:** Points of difference between GIST and pancreatic pseudocyst

Parameter	GIST	Pancreatic pseudocyst
Clinical presentation	Indolent presentation along with constitutional symptoms and symptoms based on presence/absence of metastasis.	Usually preceded by recurrent attacks of pain in abdomen associated with a lump in abdomen.
Radiological findings	Usually predominantly solid lesions with small cystic areas. Most common site of origin is gastric followed by small intestine. Multiloculated/multiseptate collection with solid component. Occasional cystic variant may present which may mimic pseudocyst of pancreas/pancreatic cystic neoplasms.	Usually a predominantly cystic lesion with peripheral enhancement. Arises from the pancreas with/without communication with the MPD.
Nature of fluid	Usually hemorrhagic with elevated CEA levels.	Usually dark brown with occasional necrotic debris. Elevated fluid amylase levels. Negative for CEA/CA 19.9.
Management	Usually surgical followed by adjuvant chemotherapy/radiotherapy depending upon the stage of the tumor.	Endoscopic internal drainage/Open or laparoscopic external drainage.

Surgery constitutes the first line of management in cases of GIST with predominantly cystic architecture. Xue et al. demonstrated in their study that surgery is a safe and efficacious alternative for patients with cystic variants of GIST (cGIST) [20]. cGIST is associated with a relatively higher risk of recurrence, based on the modified NIH-Fetcher classification system. Imatinib mesylate-based chemotherapy forms the cornerstone of adjuvant therapy in cases of GIST with a high risk of recurrence. However, owing to the higher risk of rupture and the lack of exact pathology, pre-operative imatinib administration is of questionable value. Adjuvant Imatinib therapy coupled with surgical resection forms the mainstay of management in cases of cGIST with low-grade malignancy. Radiotherapy has a definitive role in the adjuvant management of GIST. However, with cGIST, its role has not yet been proven. A five-year disease-free survival (DFS) of 60% has been reported with the use of surgery followed by adjuvant imatinib therapy in cases with cGIST [18,19].

## Conclusions

This case emphasizes the critical need for clinicians to recognize and consider atypical presentations of gastrointestinal stromal tumors (GIST), particularly those presenting with cystic changes. These variations in presentation can obscure the diagnosis, as seen in this instance, where a large cystic lesion originating from the stomach was initially mistaken for a pancreatic pseudocyst. Early recognition through thorough clinical evaluation and precise histopathological assessment following surgical resection proved pivotal in accurately diagnosing a malignant epithelioid GIST. Such cases underscore the diagnostic challenges posed by GISTs and highlight the necessity for comprehensive diagnostic approaches to ensure appropriate management and favorable patient outcomes.

## References

[1] Miettinen M, Lasota J (2011). Histopathology of gastrointestinal stromal tumor. J Surg Oncol.

[2] Yoshizawa JI, Shimizu T, Ikehara T, Fukushima K, Nakayama A (2022). Gastrointestinal stromal tumor of the small bowel complicated by torsion: A case report. Int J Surg Case Rep.

[3] Gheorghe G, Bacalbasa N, Ceobanu G (2021). Gastrointestinal stromal tumors—a mini review. J Pers Med.

[4] Sorour MA, Kassem MI, Ghazal Ael-H, El-Riwini MT, Abu Nasr A (2014). Gastrointestinal stromal tumors (GIST) related emergencies. Int J Surg.

[5] Martin-Broto J, Martinez-Marín V, Serrano C (2017). Gastrointestinal stromal tumors (GISTs): SEAP-SEOM consensus on pathologic and molecular diagnosis. Clin Transl Oncol.

[6] Sun KK, Xu S, Chen J, Liu G, Shen X, Wu X (2016). Atypical presentation of a gastric stromal tumor masquerading as a giant intraabdominal cyst: A case report. Oncol Lett.

[7] Sandrasegaran K, Rajesh A, Rydberg J, Rushing DA, Akisik FM, Henley JD (2005). Gastrointestinal stromal tumors: clinical, radiologic, and pathologic features. AJR Am J Roentgenol.

[8] Chen L, Gu J, Zhang X, Yu A (2022). Case report: Giant cystic ileal gastrointestinal stromal tumor with an atypical intratumoral abscess. Front Surg.

[9] Hamza AM, Ayyash EH, Alzafiri R, Francis I, Asfar S (2016). Gastrointestinal stromal tumour masquerading as a cyst in the lesser sac. BMJ Case Rep.

[10] Okano H, Tochio T, Suga D (2015). A case of a stomach gastrointestinal stromal tumor with extremely predominant cystic formation. Clin J Gastroenterol.

[11] Shaikh ST, Upwanshi MH, Shetty TS, Ghetla SR, Gheewala H (2015). A large cystic variant of gastro-intestinal stromal tumour arising from the Jejunum: a case report. J Clin Diagn Res.

[12] Bechtold RE, Chen MY, Stanton CA, Savage PD, Levine EA (2003). Cystic changes in hepatic and peritoneal metastases from gastrointestinal stromal tumors treated with Gleevec. Abdom Imaging.

[13] Sanchez-Hidalgo JM, Duran-Martinez M, Molero-Payan R (2018). Gastrointestinal stromal tumors: A multidisciplinary challenge. World J Gastroenterol.

[14] Wang L, Liu L, Liu Z, Tian Y, Lin Z (2017). Giant gastrointestinal stromal tumor with predominantly cystic changes: a case report and literature review. World J Surg Oncol.

[15] Miettinen M, Kopczynski J, Makhlouf HR (2003). Gastrointestinal stromal tumors, intramural leiomyomas, and leiomyosarcomas in the duodenum: a clinicopathologic, immunohistochemical, and molecular genetic study of 167 cases. Am J Surg Pathol.

[16] Parab TM, DeRogatis MJ, Boaz AM (2019). Gastrointestinal stromal tumors: a comprehensive review. J Gastrointest Oncol.

[17] Miettinen M, Lasota J (2006). Gastrointestinal stromal tumors: review on morphology, molecular pathology, prognosis, and differential diagnosis. Arch Pathol Lab Med.

[18] Hirota S, Ohashi A, Nishida T (2003). Gain-of-function mutations of platelet-derived growth factor receptor alpha gene in gastrointestinal stromal tumors. Gastroenterology.

[19] Joensuu H (2006). Gastrointestinal stromal tumor (GIST). Ann Oncol.

[20] Xue A, Yuan W, Gao X (2019). Gastrointestinal stromal tumors (GISTs) with remarkable cystic change: a specific subtype of GISTs with relatively indolent behaviors and favorable prognoses. J Cancer Res Clin Oncol.

